# Human bone marrow-derived mesenchymal stem cells promote the growth and drug-resistance of diffuse large B-cell lymphoma by secreting IL-6 and elevating IL-17A levels

**DOI:** 10.1186/s13046-019-1081-7

**Published:** 2019-02-12

**Authors:** Weijie Zhong, Zhigang Zhu, Xin Xu, Hui Zhang, Huabao Xiong, Qingshan Li, Yaming Wei

**Affiliations:** 1 Department of Geriatrics, Hematology & Oncology ward, Guangzhou First People’s Hospital, School of Medicine, South China University of Technology, Guangzhou, 510180 Guangdong China; 2 Institute of Immunology and Molecular Medicine, Jining Medical University, Jinan, 272067 Shandong China; 3 Immunology Institute, Mount Sinai School of Medicine, NY10029, New York, 5674 USA; 4 Department of Hematology, Guangzhou First People’s Hospital, School of Medicine, South China University of Technology, Panfu Rd No.1, Yuexiu District, Guangzhou, 510180 Guangdong China; 5 Department of Blood Transfusion, Guangzhou First People’s Hospital, School of Medicine, South China University of Technology, Panfu Rd No.1, Yuexiu District, Guangzhou, 510180 Guangdong China

**Keywords:** Mesenchymal stem cells, Interleukin-6, Interleukin-17A, Drug-resistance, Lymphoma, large B cell, diffuse, JAK2/STAT3, PI3K/Akt

## Abstract

**Background:**

The drug-resistance and relapse of diffuse large B-cell lymphoma (DLBCL), which are related to mesenchymal stem cells (MSCs), have become increasingly common. However, the underlying mechanisms remain elusive.

**Methods:**

CCK 8 assay, colony formation assay, and xenograft mouse model were used to investigate the effects of hBMSCs on DLBCL growth. Immunohistochemistry, qRT-PCR, and ELISA were used to study the expressions of IL-6 and IL-17A. Flow cytometry was used to analyze Th17 cells and Treg cells expressions. Western blot analysis, microarray analysis, and bioinformatics analysis were used to analyze the pathways of IL-6 or IL-17A mediated DLBCL growth.

**Results:**

HBMSCs promoted DLBCL growth by secreting IL-6 in vitro and in vivo and simultaneously upregulating IL-17A in vitro. IL-6 and IL-17A synergistically promoted the growth and drug-resistance of DLBCL cells by protecting them from spontaneous or drug-induced apoptosis in vitro. IL-6 or IL-17A activated the JAK2/STAT3 pathway or upregulated cyclin D2 via activation of PI3K/Akt signaling in vitro*,* respectively.

**Conclusions:**

The present results indicated that hBMSCs might have a “dual effect” on promoting DLBCL progression and drug-resistance by secreting IL-6 and upregulating IL-17A. IL-6, IL-17A, p-STAT3, p-Akt or cyclin D2 may be potential molecular targets for overcoming drug-resistance in patients with relapsed or refractory DLBCL.

## Background

Diffuse large B-cell lymphoma (DLBCL) is the most common subtype of non-Hodgkin’s lymphoma (NHL), with an incidence of more than 100,000 cases per year worldwide [1]. Because of the clinical use of rituximab in combined immuno-chemotherapy, approximately 80% of these patients may achieve complete remission [2]. However, most of the remaining patients will develop drug-resistance and finally relapse. Approximately 30% of cases are resistant to rituximab or rituximab-based chemotherapy regimens [3, 4]. Furthermore, 60% of lymphoma patients develop acquired drug-resistance [5]. These issues are challenging for clinicians, and the mechanisms of drug-resistance and relapse of DLBCL patients remain unclear.

Increasing evidence indicates that the tumor microenvironment (TME) contributes significantly to B-cell lymphoma pathogenesis, progression, drug-resistance and metastasis [6]. In addition to tumor cells, the TME is composed of a mixture of mesenchymal stem cells (MSCs), immune cells (T, B, and dendritic cells), extracellular matrix, and blood vessels. MSCs are a heterogeneous group of fibroblast-like progenitor cells, and they are mainly derived from the bone marrow. Studies show that MSCs can migrate to tumor sites and play a crucial role in promoting lymphoma growth, survival, metastasis, and drug-resistance [7, 8]. Human bone marrow derived MSCs (hBMSCs) promote the growth of mantle cell lymphoma (MCL) cells [9] and DLBCL cells [10] by protecting them from spontaneous and drug-induced apoptosis. Nevertheless, the mechanism by which hBMSCs promote DLBCL growth and drug-resistance remains elusive.

Interleukin (IL)-6, which is one of the most important cytokines secreted by MSCs in the TME, plays an important role in regulating the immune response, tumor cell proliferation, tumor cell apoptosis, and tumorigenesis [11]. Studies show that IL-6 activates the JAK2/STAT3 and Akt signaling pathways to promote growth and induce drug-resistance in tumors including MCL [11, 12]. High levels of IL-6 in the TME of MCL promote tumor growth and induce drug-resistance [12]. Meanwhile, IL-6 levels are significantly increased in the peripheral blood of DLBCL patients and indicate a poor prognosis [13, 14].

Many immune cells infiltrate the TME. T helper (Th) 17 and regulatory T (Treg) cells are two types of CD4+ T cells that play a critical role in promoting the tumorigenesis and progression of NHL. Th17 cells mainly secrete IL-17A, a member of the IL-17 family (IL-17A–F), and the process is enhanced by IL-6 combined with transforming growth factor (TGF)-β [15]. Treg cells mainly secrete TGF-β and IL-10. A previous study showed that IL-17A promotes the growth of human germinal center-derived NHL, including DLBCL [16]. In previous studies, we demonstrated that irradiated or rituximab-treated NHL cells (k1106 cells or SU-DHL-4 cells) induce Foxp3+ Treg and Th17 cells to secrete IL-17 by increasing the secretion of IL-6; the secreted IL-17 then inhibits the irradiation-induced apoptosis of NHL cells by suppressing p53 [17–19]. Therefore, IL-17A is a pro-tumorigenic factor in DLBCL. MSCs have a powerful function in immune regulation, while hBMSCs promote Th17 cell expansion and increase IL-17A levels in rheumatoid arthritis in vitro [20]. Furthermore, BMSCs promote malignant B cell tissue homing and development by modulating the stromal cell-derived factor-1(SDF-1)/chemokine (C-X-C motif) receptor 4 (CXCR4) axis [21]. However, the mechanisms by which hBMSCs promote DLBCL growth and drug-resistance remain unclear.

Based on previous data, we hypothesized that hBMSCs may promote DLBCL growth and drug-resistance by simultaneously secreting IL-6 and increasing IL-17A levels in the TME. In the present study, we investigated the effects of hBMSCs on the growth and drug-resistance of DLBCL in vitro and in vivo in the presence or absence of peripheral blood mononuclear cells (PBMCs). We demonstrated that hBMSCs secrete IL-6 in vitro and in vivo and play a role in the differentiation of Th17 and Treg cells, thereby modulating IL-17A and TGF-β levels in the TME of DLBCL cells in vitro. The synergistic effects of IL-6 and IL-17A on inhibiting the spontaneous or drug-induced apoptosis of DLBCL cells were verified in vitro. Finally, the signaling pathways mediating the effects of IL-6 or IL-17A on promoting DLBCL cell growth were analyzed by microarray analysis, western blotting, and qPCR.

## Methods

### Reagents

Cell Counting Kit-8 (CCK-8) was purchased from Dojindo Laboratories (Kumamoto, Japan). Human recombinant IL-6 and IL-17A, and human neutralizing antibodies to IL-6 (aIL-6) and IL-17A (aIL-17A) were obtained from R&D Systems (Minneapolis, MN, USA). The monoclonal mouse anti-human IL-6 antibody (ab9324) was purchased from Abcam (Shanghai, China). Enzyme-linked immunosorbent assay (ELISA) kits for IL-17A, IL-6, IL-10, IL-1β, PGE_2_, and TGF-β; fluorescence-activated cell sorting (FACS) human antibodies including anti-CD4-FITC, anti-IL-17A-APC, anti-Foxp3-PE, anti-CD25-APC, and their corresponding anti-mouse IgG1 K-PE/APC; and the Annexin V apoptosis detection kit were all purchased from eBioscience (San Diego, CA, USA). Antibodies against JAK2, p-JAK2, STAT3, p-STAT3, p-AKT, AKT, cyclin D2, P27, and *GAPDH* were purchased from Santa Cruz Biotech (Santa Cruz, CA, USA). Real-time reverse transcription-polymerase chain reaction (RT-PCR) reagents were obtained from Takara (Beijing, China).Rituximab was purchased from Novartis (Basel, Switzerland). Doxorubicin and Ara-C were obtained from Pfizer (Shanghai, China).

### Human samples and cell lines

We collected 48 paraffin-embedded tumor specimens from DLBCL patients and 18 paraffin-embedded benign lymph node specimens from acute lymphadenitis patients at Guangzhou First People’s Hospital, between 2010 and 2016. The clinical characteristics of the patients are shown in Table 1. All DLBCL patients were diagnosed by experienced pathologists and were consistent with DLBCL diagnostic criteria. PBMCs were isolated from blood samples of healthy volunteers using the Ficoll–Hypaque method. PBMCs were cultured in RPMI1640 medium (Gibco, New York, USA) containing 100 U/mL penicillin (Gibco), 100 U/mL streptomycin (Gibco), and 10% fetal bovine serum (FBS) (Gibco). This research was approved by the Ethics Committee of Guangzhou First People’s Hospital (K-2017-066-02). Written informed consent was obtained from all participants or their families. The SU-DHL-2 and SU-DHL-4 cell lines were purchased from ATCC (Shanghai, China) and cultured in RPMI 1640 medium containing 10% FBS, 4 mM L-glutamine (Gibco), 100 U/ml of penicillin, and 100 U/ml of streptomycin. HBMSCs were purchased from Cyagen Biosciences (Santa Clara, CA, USA) and cultured in OriCell™ hBMSCs complete medium (Cyagen Biosciences). All cells were cultured in a humidified chamber at 37 °C with an atmosphere of 5% CO_2_.

**Table 1:** Clinical characteristics of 48 DLBCL patients

Characteristics	No. (%)
Age	
Median	55
Range	32–75
Gender	
Male	30 (62.5)
Female	18 (37.5)
Ann Arbor stage^a^	
I-II	10 (20.8)
III-IV	38 (79.2)
IPI score^b^	
1–3	37 (77.1)
4–5	11 (22.9)

^a^Ann Arbor stage according to Ann Arbor-Cotswald stage (1989)

^b^IPI score, International Prognostic Index score

### In vitro cell culture

Four in vitro cell culture experiments were performed. All experiments were performed using SU-DHL-2/4 cells and PBMCs at 1 × 10^6^ cells/well.

Experiment 1: SU-DHL-2/4 cells were cultured with or without hBMSCs at different ratios of 1:1, 1:5, and 1:0.2 in 6-well plates with or without Transwell inserts (CORNING, New York, USA), and then incubated for 72 h. The proliferation of SU-DHL-2/4 cells was analyzed using the CCK-8 assay, and the supernatants were detected by ELISA.

Experiment 2: SU-DHL-2/4 cells were cultured with or without PBMCs and/or hBMSCs at the ratio of 1:1:1 in 6-well plates with Transwell inserts. SU-DHL-2/4 cells were placed in the inserts and the other cells were seeded in the outer wells. All groups were incubated for 72 h and then detected using the CCK-8 assay, colony formation assay, and ELISA.

Experiment 3: Eight groups of cells were cultured with or without Transwell inserts for 72 h as follows: PBMCs alone; PBMCs and hBMSCs; PBMCs and SU-DHL-2 cells; PBMCs, SU-DHL-2 cells, and hBMSCs; PBMCs and SU-DHL-4 cells; PBMCs, SU-DHL-4 cells, and hBMSCs; SU-DHL-2 cells and hBMSCs; SU-DHL-4 cells and hBMSCs. The culture ratio was 1:1:1. After incubation, PBMCs were detected by FACS and qPCR, and the supernatants were detected by ELISA.

Experiment 4: SU-DHL-2/4 cells were co-cultured with or without hBMSCs (1:1), IL-6 (0.5 ng/ml), aIL-6 (50 μg/ml), IL-17A (0.1 ng/ml) and/or aIL-17A (10 μg/ml) for 72 h, and rituximab (10 μg/ml), doxorubicin (2 μM) and Ara-C (2 μM) were added 24 h before detection to induce apoptosis. The SU-DHL-2/4 cells were then collected and analyzed using CCK-8 and apoptosis assays.

### Cell viability and colony formation assay

The viability of SU-DHL-2/4 cells was assessed using the CCK-8 assay. Briefly, each sample was allocated in 96-well plates, and CCK-8 was added 4 h before the end of the culture time. The absorbance (optical density, OD) at 450 nm was measured at different time points using a Universal Microplate Spectrophotometer (Thermo Fisher Scientific, Inc., Waltham, MA, USA). The soft agar colony formation assay (CFA) was performed using 24-well plates according to a previous study [22]. The cells were cultured in a cell incubator for 2 to 3 weeks, and then fixed with 10% paraformaldehyde and stained with 1% crystal violet in 70% ethanol. Colonies (with minimal 50 cells) were counted using a stereomicroscope.

### Immunohistochemistry

The expression of IL-6 in the 48 human tumor tissues, 18 human benign tissues, and 24 mice tumor tissues was detected by immunohistochemistry **(**IHC). Tissues were fixed in 10% neutral formaldehyde, embedded in paraffin, sliced and stained with hematoxylin and eosin. Briefly, the paraffin-embedded tissues were serially cut into 4 μm sections, dewaxed, and rehydrated. Sections were then blocked with peroxide and non-immune animal serum and incubated sequentially with primary antibody, biotin-labeled secondary antibody, and streptomycin anti-biotin peroxidase. Finally, the sections were stained with di-n-butyl adipate, counterstained with hematoxylin, dehydrated, cleared in xylene, and fixed. The expression of IL-6 in paraffin sections was quantified by relative integrated optical density (IOD). At least three different images of each paraffin section were acquired at × 40 high power microscopic fields, and the IOD of images and mean IOD of each paraffin section were calculated by experienced pathologists using Image-Pro-Plus 6.0 software (Media Cybernetics, Rockville, MD, USA). The paraffin section with the lowest mean IOD was used as the negative control, and the relative IOD was calculated as the mean IOD/negative control.

### qRT-PCR

Total RNA was isolated from PBMCs, SU-DHL-2 cells or mice tumor tissues using the Trizol reagent (Takara) according to the manufacturer’s instructions. RNA was reverse-transcribed into cDNA using PrimeScript RT Master Mix (Takara) according to the manufacturer’s protocols. qPCR was performed using SYBR Premix Ex TaqII (TliRNaseH Plus) (Takara) on a Light Cycler 480II system (Roche, Mannheim, Germany). The levels of IL-17A, IL-10, TGF-β, RORγt, Foxp3, IL-6, CSF1, CCND2, CDKN1B, EFNA3, FGFR2, FGFR3, IL2RB, and ITGA9 were normalized to that of glyceraldehyde-3-phosphate dehydrogenase (GAPDH). The primers (5′–3′) used for qPCR are presented in Table 2.

**Table 2:** The primers (5′–3′) used for qPCR

Gene name	Direction	Sequence
IL-17A	Forward	CTGCTACTGCTGCTGAGCCTG
	Reverse	GGTTATGGATGTTCAGGTTGACC
IL-10	Forward	CTCAGCACTGCTCTGTTGCC
	Reverse	TCTCGGAGATCTCGAAGCATG
TGF-β	Forward	GACCTCTTGGCGCGACG
	Reverse	GGCTGGTCCGGAATGGG
RORγt	Forward	GCAGAGCTTCAGGCTGAGGC
	Reverse	CATGACTGAGCCTTGGCTCTG
Foxp3	Forward	GCCCTTGGACAAGGACCC
	Reverse	CAGCAGGTCTGAGGCTTTGG
IL-6	Forward	CTGCAAGAGACTTCCATCCAG
	Reverse	AGTGGTATAGACAGGTCTGTTGG
CSF1	Forward	CTCGGCTCACCTAAGTGCC
	Reverse	CAGAGGCCAGTGCTTGATCC
CCND2	Forward	GTACTGGAGGCTCTGTTCTGCC
	Reverse	GAAGCGCAGCTCCGTCTG
CDKN1B	Forward	CTGAGGAACTGACGTGGAGC
	Reverse	GAAGTATCAGCTGTCTCTGAAAGGG
EFNA3	Forward	AGAGAACCCTCAGGTGCCC
	Reverse	CATGAGGAAGAAGGCGATGC
FGFR2	Forward	ACCTAGCTACACTGAGCAGGGAG
	Reverse	GGTGGCTTGTGGCAGTCC
FGFR3	Forward	CAGGTGCAGAGGTACCCTGG
	Reverse	GTGGTGCAAAGGCAGAGGC
ITGA9	Forward	GAACTCTGAACTTTGGAGAGTGAGC
	Reverse	AGGCCAGATATCAGTGCTTGAGTG
IL2RB	Forward	CTTCTCCTGGAGGGAAGCAC
	Reverse	CACTCTGAGGCCTCAGAGATCC
*GAPDH*	Forward	GCACCGTCAAGGCTGAGAAC
	Reverse	TGGTGAAGACGCCAGTGGA

### Western blot analysis

SU-DHL-2/4 cells or tumor tissues were lysed using sodium dodecyl sulfate buffer containing proteinase inhibitors (Roche). Equal amounts of protein (50 μg) were separated by 10% sodium dodecyl sulfate-polyacrylamide gel electrophoresis and transferred onto polyvinylidene difluoride membranes (Bio-Rad, Shanghai, China). The membranes were blocked and incubated with specific antibodies overnight at 4 °C. The antibodies used were JAK2, p-JAK2, STAT3, p-STAT3, p-Akt, Akt, cyclin D2, P27, IL-6, and *GAPDH*. The membranes were then incubated with horseradish peroxidase-labeled secondary antibody (Santa Cruz Biotechnology). The protein bands were visualized using an enhanced chemiluminescence reagent.

### Flow cytometry

The frequencies of Th17 cells and Treg cells were detected by flow cytometry. Cell density was adjusted to 2 × 10^6^/ml. For Th17 cells, cells were stimulated by the addition of 50 ng/ml phorbol myristate acetate, 1 μg/ml ionomycin, and 10 μg/ml Brefeldin A to the medium for 5 h at 37 °C and 5% CO_2_. The cells were then stained with anti-CD4-FITC and anti-IL-17A-APC antibodies for 1 h at room temperature. For Treg cells, cells were stained with anti-CD4-FITC, anti-Foxp3-PE, and anti-CD25-APC antibodies for 1 h at room temperature. The cells were analyzed with a flow cytometer (FACS Canto II; BD Bioscience) and the data were analyzed with FlowJo 10 software. CD4 + IL-17A+ cells were defined as Th17 cells, and CD4 + CD25 + Foxp3+ cells were defined as Treg cells.

### Elisa

Cell culture supernatants were collected at the indicated times, and then supernatants were assessed for IL-17A, IL-6, IL-10, IL-1β, PGE_2,_ and TGF-β by ELISA, following the manufacturer’s instructions.

### Apoptosis assay

The Annexin V/propidium iodide assay was used to detect DLBCL cell apoptosis. Cells were seeded in 48-well plates and incubated with or without chemotherapy drugs for 72 h. After culture, cells were washed twice with cold PBS and resuspended in binding buffer at a concentration of 1 × 10^6^ cells/ml after which 100 μL of the solution was transferred to a 5-mL tube, and 5 μL of Annexin V–FITC and 5 μL of propidium iodide were added. The tube was gently vortexed and incubated for 15 min at room temperature in the dark. At the end of incubation, 300 μL of binding buffer was added. Flow cytometric analysis was performed immediately with a flow cytometer (FACS Canto II; BD Bioscience).

### Human expression microarray analysis and bioinformatics analysis

SU-DHL-2 cells (2 × 10^6^ cells/ml) co-cultured with or without IL-17A (200 pg/ml) for 72 h were harvested, and the total RNA of each sample was extracted using the TRIzol reagent (Takara).The Whole Human Genome Oligo Microarray (4 × 44 K, Agilent Technologies) was generated by KangChen Biotechnology. Briefly, total RNA from each sample was linearly amplified and labeled with Cy3-UTP. The Labeled cRNAs were purified using the RNeasy Mini Kit (Qiagen, Hilden, Germany). The concentration and specific activity of the labeled cRNAs (pmol Cy3/μg cRNA) were measured by NanoDrop ND-1000 (Thermo). Aliquots of 1 μg of each labeled cRNA were fragmented by adding 11 μl of 10 × Blocking Agent and 2.2 μl of 25 × Fragmentation Buffer, then heated at 60 °C for 30 min, and finally 55 μl of 2 × GE Hybridization buffer was added to dilute the labeled cRNA. One-hundred microliters of hybridization solution were dispensed into the gasket slide and assembled to the gene expression microarray slide. The slides were incubated for 17 h at 65 °C in an Agilent Hybridization Oven. The hybridized arrays were washed, fixed, and scanned using the Agilent DNA Microarray Scanner (part number G2505C). Array images were analyzed using Agilent Feature Extraction software (version 11.0.1.1). Quantile normalization and subsequent data processing were performed with the GeneSpring GX v12.1 software package (Agilent Technologies). After quantile normalization of the raw data, genes for which at least 3 out of 6 samples had flags in Detected (“All Targets Value”) were chosen for further data analysis. Differentially expressed genes (DEGs) with statistical significance between the two groups were identified through Volcano Plot filtering. DEGs between the two samples were identified through Fold Change filtering (fold change ≥1.5, *P* < 0.05). Hierarchical clustering and heat map were performed using the R scripts. The present microarray data is accessible through Gene Expression Omnibus, series accession number GSE115855 (https://www.ncbi.nlm.nih.gov/geo/query/acc.cgi?acc=GSE115855). Gene ontology (GO) analysis and pathway analysis were based on the GO database (http://www.geneontology.org) and KEGG database (http://www.kegg.jp). Geneco-expression networks, in which the core genes that play a vital role in the network were identified, were constructed based on the STRING database (https://string-db.org/) and further analyzed by Cytoscape software (version 3.6.0). In the networks, nodes were mainly genes and edges represented relation types between the nodes. Node size represented the degree of expression of each gene: green and red nodes represented down- and up-regulated genes, and edge size represented the combined score.

### Xenograft mouse model

Male nude mice (BALB/c, 20–30 g) (Guangdong Laboratory Animal Center, China) 6–8 weeks of age were used in experiments. Mice were housed and monitored in micro-isolator cages under clean conditions. All experimental procedures and protocols were approved by the Institutional Animal Care and Use Committee at Guangzhou First People’s Hospital. SU-DHL-4 cells (1 × 10^7^cells/100 μL PBS) were subcutaneously inoculated into the right flanks of the nude mice. After the development of 3 mm diameter tumors, mice were randomly assigned to three groups (*n* = 8 per group). The MSC group was injected with circumtumoral hBMSCs (5 × 10^5^ cells/100 μL PBS per time); the IL-6 group was injected with circumtumoral IL-6 (10 μg/kg per time); and the Control group was injected with circumtumoral PBS (100 μL per time). All groups were injected five times every 3 days (on days 0, 3, 6, 9, and 12). The size of tumors was measured every 3 days, up to 28 days following hBMSC, IL-6, and PBS injection. Tumor volume was calculated according to the following formula: tumor volume (mm^3^) = (a^2^ × b)/2, where “a” represents the length of the short axis and “b” represents the length of the long axis. The mice were sacrificed 28 days after hBMSC, IL-6, and PBS injection, and the tumors were removed and prepared for IHC, qPCR, and western blot analysis.

### Statistical analysis

For microarray analysis, DEGs were confirmed using a *p*-value threshold and false discovery rate (FDR) analysis. The threshold for truly significant genes was determined at a *p*-value of < 0.05 and FDR value < 0.05. All analyses were performed using SPSS 17.0. Numerical data were presented as the mean ± standard deviation. Two-tailed independent-sample Student’s t/t′ tests were used for comparisons between two groups. Single-factor analysis of variance (one-way ANOVA) and Student–Newman–Keuls/Dunnett’s T3 tests (between every two groups) were used for comparisons among multiple groups. The Chi-square test was used for enumeration data comparisons between two groups. A *p*-value of 0.05 was considered significant.

## Results

### hBMSCs promote the growth of DLBCL cells in vitro, and PBMCs enhance these effects

**Fig. 1:**
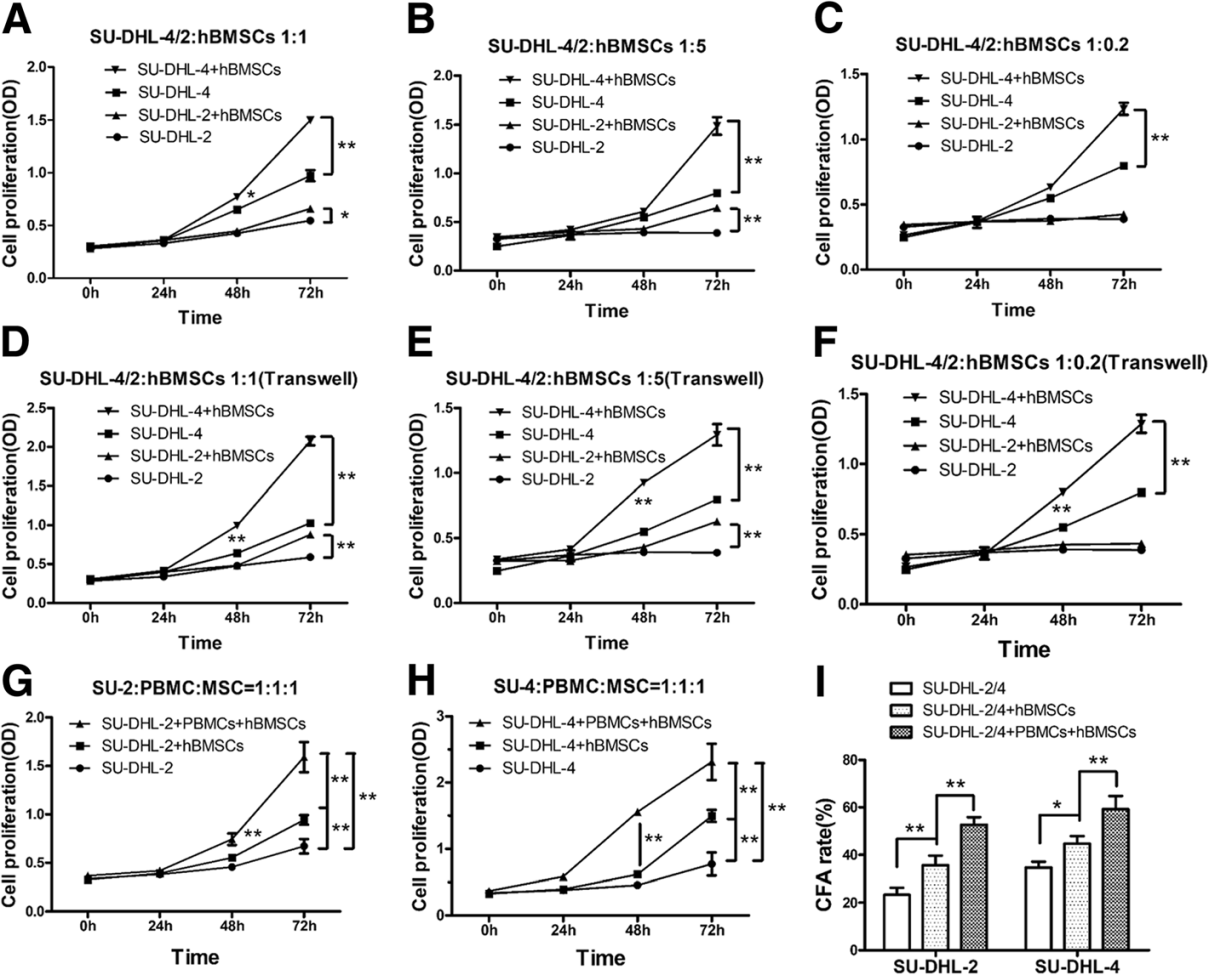
hBMSCs promoted the growth of DLBCL cells in vitro, and PBMCs enhanced these effects. DLBCL cells (SU-DHL-2 and SU-DHL-4 cells) were co-cultured with hBMSCs at different ratios of 1:1, 1:5, or 1:0.2 in direct co-culture systems for 72 h. **a**, **b**, and **c**: Graphs of DLBCL cell proliferation (OD) detected by CCK-8 assay in direct co-culture systems. **d**, **e**, and **f**: Graphs of DLBCL cell proliferation (OD) in indirect co-culture systems. PBMCs were added to a co-culture with DLBCL cells and hBMSCs at a 1:1:1 ratio for 72 h. **g** and **h**: Graphs of DLBCL cell proliferation (OD). **i**: CFA rates of DLBCL cells co-cultured for 72 h. The data shown represent one of three independent experiments. Error bars represent standard deviation (SD). Significance was determined using single-factor analysis of variance (one-way ANOVA) (three groups) or two-tailed independent-sample Student’s t/t′ test (two groups). (*P < 0.05; **P < 0.01)

To investigate whether hBMSCs promote the growth of DLBCL cells in vitro, hBMSCs were co-cultured with SU-DHL-2 or SU-DHL-4 cells at different ratios in a direct or indirect system for 72 h. Then, SU-DHL-2 and SU-DHL-4 cell proliferation was detected using the CCK-8 assay at different time points. HBMSCs significantly increased the proliferation of SU-DHL-4 cells at ratios of SU-DHL-4:hBMSC of 1:1, 1:5, or 1:0.2 in a time-dependent manner in the direct (Fig. 1a–c) or indirect (Fig. 1d–f) co-culture system. Similarly, hBMSCs markedly promoted the proliferation of SU-DHL-2 cells at SU-DHL-2:hBMSC ratios of 1:1 and 1:5 directly (Fig. 1a and b) or indirectly (Fig. 1d and e). However, hBMSCs had no effect at a ratio of 1:0.2 (Fig. 1c and f). These results suggested that hBMSCs promoted the proliferation of DLBCL cells by secreting soluble cytokines instead of cell-to-cell contact.

In addition to MSCs, many immune cells that infiltrate the TME of DLBCL can affect the growth of tumor cells and the promoting effects of hBMSCs. To simulate the TME of DLBCL, SU-DHL-2 and SU-DHL-4 cells were co-cultured with hBMSCs and PBMCs at a ratio of 1:1:1 for 72 h, and the cells were separated using Transwell inserts. The proliferation and clonogenicity of DLBCL cell lines were tested using the CCK-8 assay and CFA, respectively.. PBMCs significantly increased the effect of hBMSCs on promoting the proliferation of SU-DHL-2 and SU-DHL-4 cells (Fig. 1g and h). Furthermore, hBMSCs accelerated the clonogenicity of SU-DHL-2 and SU-DHL-4 cells, and PBMCs increased these effects (Fig. 1i). According to these data, we hypothesized that hBMSCs might promote the growth of DLBCL cells not only directly through the secretion of paracrine soluble cytokines, but also by affecting PBMC differentiation, as differentiated PBMCs secreted cytokines that enhanced these promoting effects.

### hBMSCs secrete IL-6 into the co-culture supernatants, and the IL-6 level is higher in DLBCL tumor tissue than in benign tissue

**Fig. 2:**
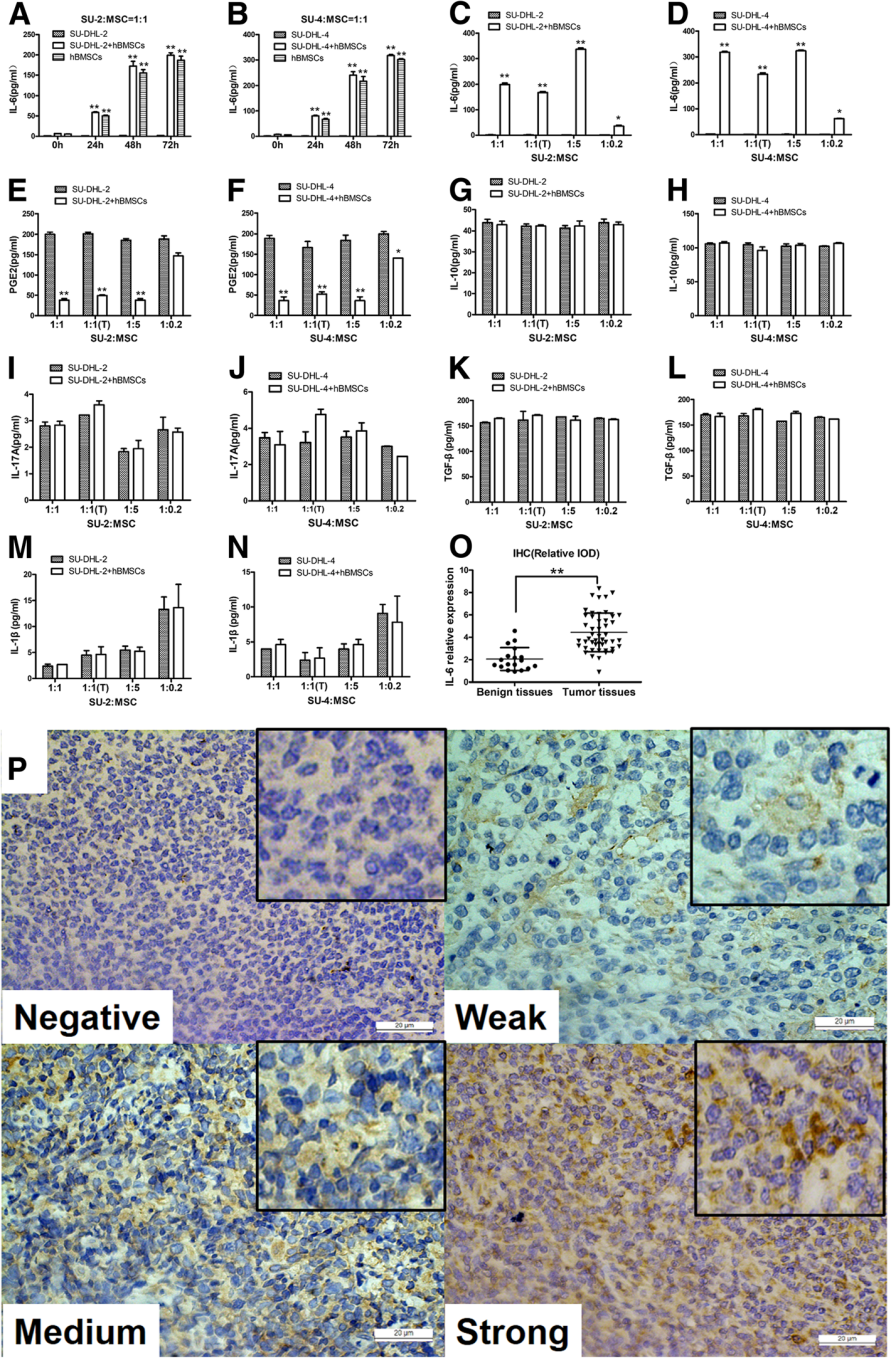
hBMSCs secreted IL-6 into the co-culture supernatants and the IL-6 level was higher in the DLBCL tumor microenvironment. **a** and **b**: Levels of IL-6 at different time points in the 1:1 co-culture supernatants of SU-DHL-2/4 cells alone, SU-DHL-2/4 cells with hBMSCs, or hBMSCs alone. Levels of IL-6 (**c** and **d**), PGE2 (**e** and **f**), IL-10 (**g** and **h**), IL-17A (**i** and **j**), TGF-β (**k** and **l**), and IL-1β (**m** and **n**) in the 72 h direct or indirect co-culture supernatants of SU-DHL-2/4 cells with or without hBMSCs at different ratios. **o**: Graph of IL-6 relative expression (relative IOD) detected by IHC from benign tissues (*n* = 48) and tumor tissues (*n* = 18). **p** IHC staining showed various intensities of IL-6 expression in the intercellular spaces of tumor cells or tumor infiltrating lymphocytes (× 400). The data shown represent one of three independent experiments. Error bars represent SD. Significance was determined using two-tailed independent-sample Student’s t/t′ test. (*P < 0.05; **P < 0.01, compared with the SU-DHL-2/4 group)

The results of the present study showed that hBMSCs promoted the growth of DLBCL cells by secreting soluble cytokines; therefore, we attempted to identify these cytokines. The results showed that hBMSCs strongly secreted IL-6 into the co-culture supernatant in a time-dependent manner when co-cultured with or without SU-DHL-2 and SU-DHL-4 cells (Fig. 2a and b).In addition, hBMSCs markedly secreted IL-6 at different ratios in a direct or indirect co-culture system (Fig. 2c and d). The level of PGE2 was decreased in the hBMSC groups at different ratios (Fig. 2e and f). The levels of IL-10, IL-17A, TGF-β, and IL-1-β did not differ between the two groups (Fig. 2g–n). To further verify the IL-6 level in the TME of DLBCL, the relative expression ofIL-6 was examined in tumor tissues and benign tissues by IHC. IL-6 levels were markedly higher in tumor tissues than in benign tissues (Fig. 2o and p). These results indicated that hBMSCs secreted IL-6 into the TME of DLBCL, thereby promoting the growth of DLBCL.

### hBMSCs promote DLBCL growth by secreting IL-6 in vivo

**Fig. 3:**
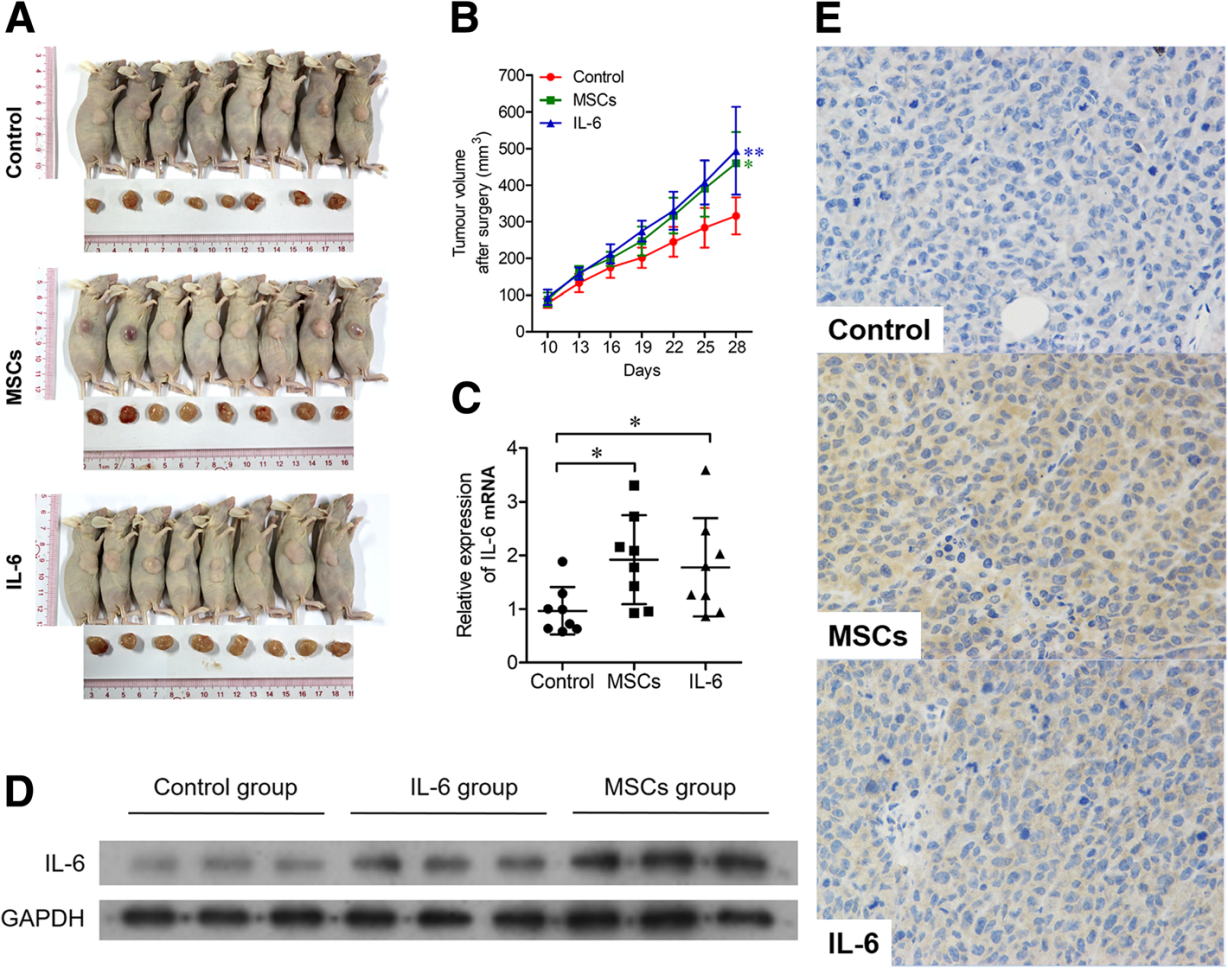
hBMSCs enhanced the growth of DLBCL by secreting IL-6 in vivo. SU-DHL-4 cells were injected subcutaneously into the right flanks of nude mice (BALB/c, *n* = 24). After the xenograft mouse models were established, hBMSCs (MSCs group, *n* = 8), IL-6 (IL-6 group, *n* = 8) or PBS (Control group, *n* = 8) were injected around the tumors. **a**: The xenograft mice were killed and the tumor tissues were removed at 28 days after hBMSC, IL-6, or PBS injection. **b**: Tumor volume, (**c**): relative expression of IL-6 mRNA and (**d**): IL-6 protein expression were measured and detected in each group. **e**: Representative IHC staining images showing various intensities of IL-6 expression in the intercellular spaces of tumor cells or tumor infiltrating lymphocytes (× 400) in each group. Error bars represent SD. Significance was determined using one-way ANOVA. (*P < 0.05; **P < 0.01)

As hBMSCs promoted the growth of DLBCL cells in vitro, we investigated whether hBMSCs have a similar effect in vivo. Because SU-DHL-4 cells showed a high rate of proliferation (Fig. 1c-h), this cell line was used to establish DLBCL xenograft BALB/c nude mouse models. Tumors developed well in nude mice, and tumor tissues were obtained by operation at 28 days after hBMSC, IL-6, or PBS injection (Fig. 3a). The tumor volumes were significantly larger in the MSC and IL-6 groups than in the Control group (Fig. 3b). In addition, IL-6 mRNA and protein levels were markedly higher in the MSC and IL-6 groups than in the Control group (Fig. 3c and d). IHC staining detected higher IL-6 levels in the tumor tissues of the MSC and IL-6 groups than in controls (Fig. 3e). These results suggested that hBMSCs promoted DLBCL growth by secreting IL-6 in vivo.

### hBMSCs induce PBMCs differentiation into Th17 and Treg cells, thereby increasing IL-17A and TGF-β levels in the co-culture supernatants

**Fig. 4:**
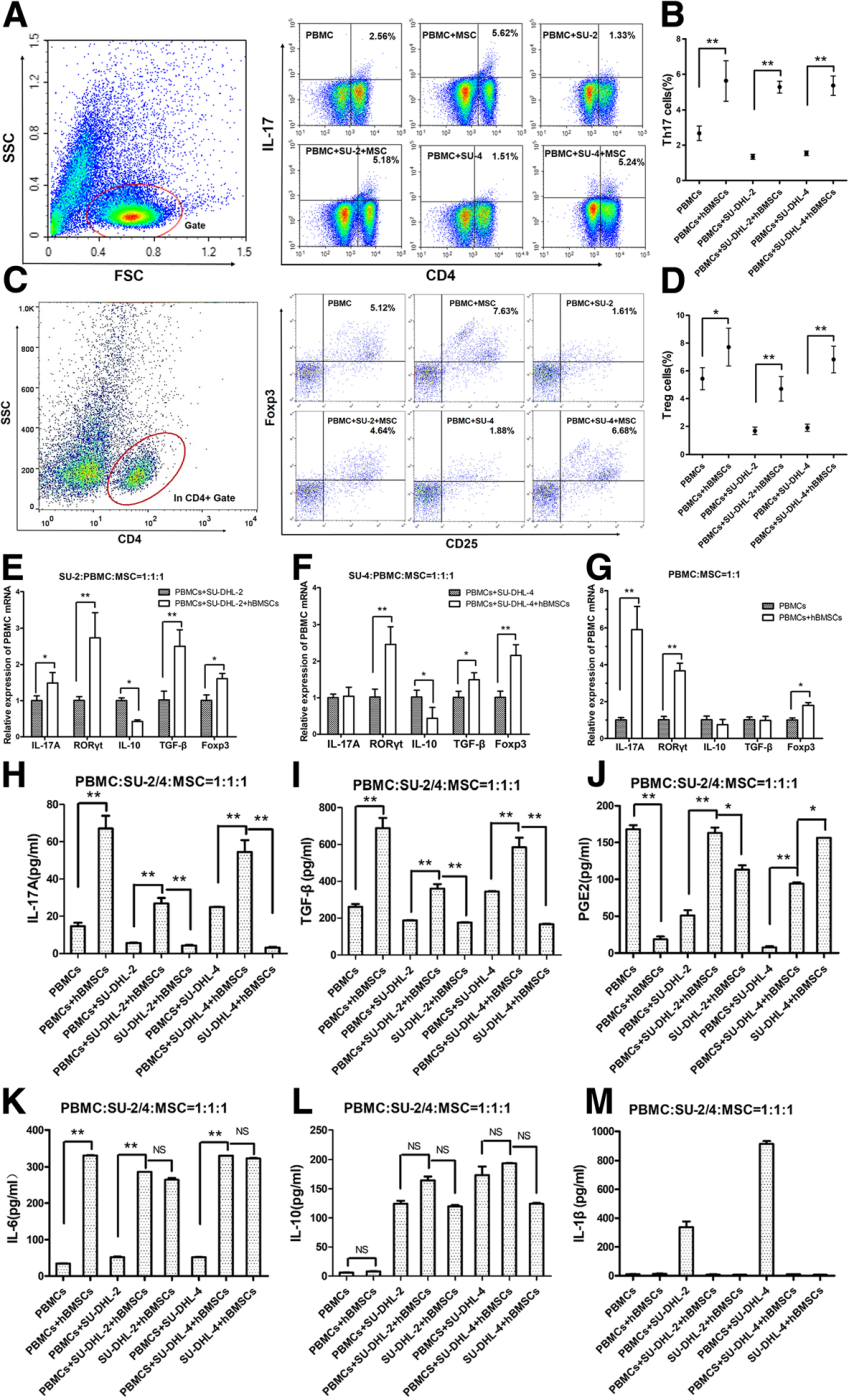
hBMSCs induced PBMCs differentiation into Th17 and Treg cells, thereby elevating IL-17A and TGF-β levels in the co-culture supernatants. PBMCs from healthy donors were divided into six groups and co-cultured with or without SU-DHL-2/4 cells and/or hBMSCs, and SU-DHL-2/4 cells with hBMSCs were selected as two control groups. All six groups containing PBMCs were co-cultured with IL-2 (100 IU/ml) for 72 h and then detected by FACS. **a** and **c** Representative FACS plots of Th17 and Treg cell percentages in PBMCs from six groups. **b** and **d** Graphs of Th17 and Treg cell mean percentages from six groups. **e**, **f**, and **g** Graphs of the mRNA levels of related cytokines and transcription factors in the PBMCs from six groups. Levels of IL-17A (**h**), TGF-β (**i**), PGE2 (**j**), IL-6 (**k**), IL-10 (**l**), and IL-1β (**m**) in the co-culture supernatants of eight groups. Cytokines were detected by ELISA. The data shown represent one of three independent experiments. Error bars represent SD. Significance was determined using one-way ANOVA. (*P < 0.05; **P < 0.01)

As PBMCs enhanced the promoting effects of hBMSCs, we next explored the underlying mechanisms. For this purpose, eight groups of co-cultures were generated using hBMSC, PBMC, or DLBCL cell lines for 72 h. The cells were separated using Transwell inserts, and the PBMCs from six groups were detected by FACS and qPCR, and the supernatants from eight groups were detected by ELISA. Comparison of three pairs of groups in the presence or absence of hBMSCs showed that hBMSCs significantly increased the frequencies of Th17 (Fig. 4a and b) and Treg (Fig. 4c and d) cells in PBMCs in each pair of groups. At the transcriptional level, hBMSCs upregulated the relative expressions of RORγt and Foxp3 mRNA in PBMCs when co-cultured with PBMCs or DLBCL cell lines (Fig. 4e–g). In addition, hBMSCs upregulated the relative expression of IL-17A and TGF-β mRNA of PBMCs (Fig. 4e–g). Similarly, hBMSCs increased the levels of IL-17A and TGF-β proteins in the supernatants of co-cultures (Fig. 4h and i). IL-6 levels were significantly higher in the groups that included hBMSCs (Fig. 4k), suggesting that hBMSC-secreted IL-6 in the supernatants induced PBMC differentiation into Th17 cells. In addition, high TGF-β levels induced Treg cell differentiation. However, hBMSCs had no consistent effects on the levels of PGE2, IL-10, and IL-1β (Fig. 4j, l, and m).

### hBMSCs or IL-6 promote the growth of DLBCL cells by protecting them from spontaneous or drug-induced apoptosis, and IL-17A reinforces these effects

**Fig. 5:**
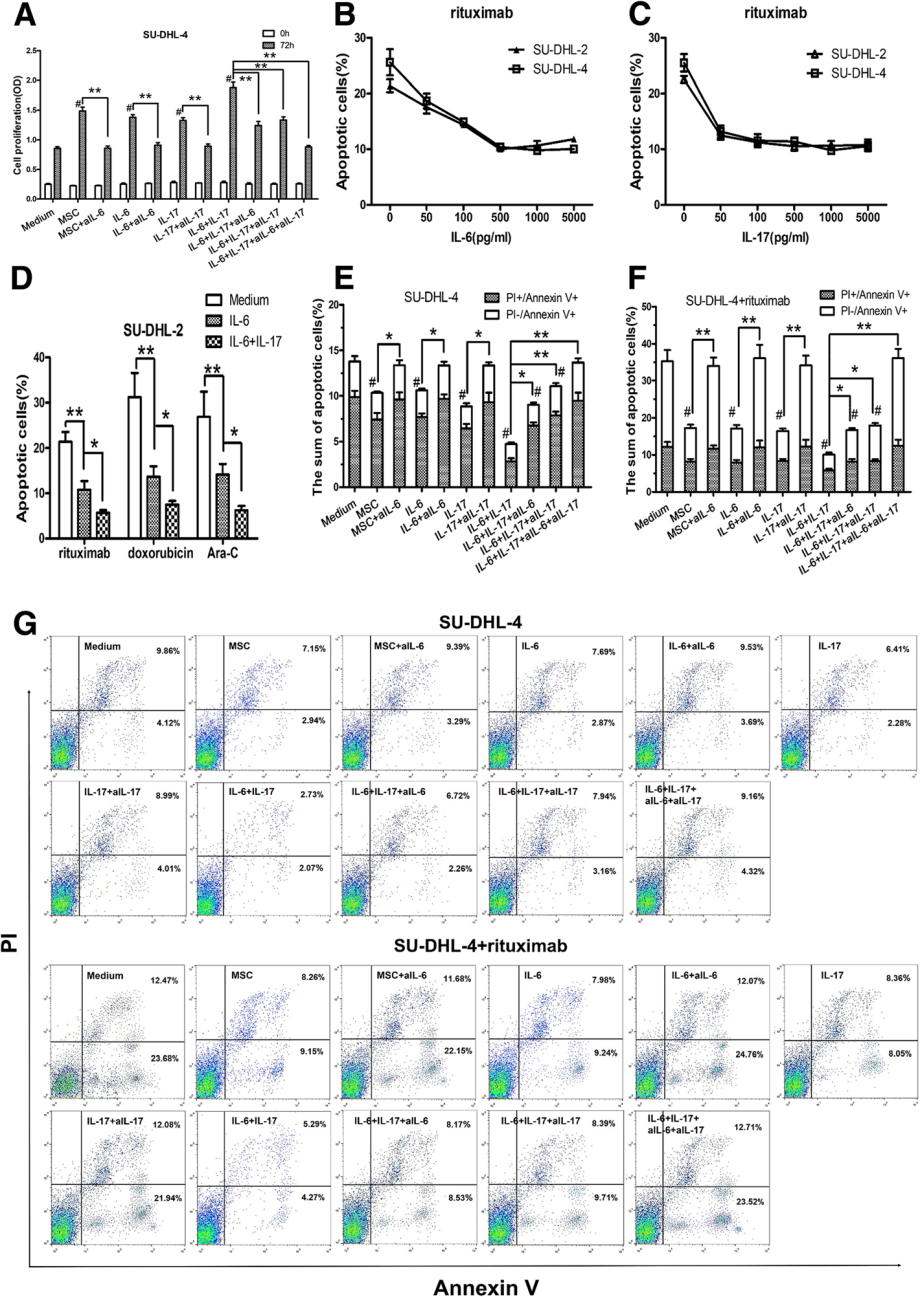
hBMSCs or IL-6 promoted the growth of DLBCL cells by protecting them from spontaneous or drug-induced apoptosis, and IL-17A reinforced these effects. **a** Graphs of cell proliferation (OD) in 72-h cultures of SU-DHL-4 cells with or without hBMSCs (1:1), exogenous IL-6 (0.5 ng/ml), IL-17A (0.1 ng/ml), aIL-6 (50 μg/ml) and/or aIL-17A (10 μg/ml). **b** and **c** Apoptosis induced by rituximab (10 μg/ml) was protected by IL-6 or IL-17A. DLBCL cell cultures were first treated with exogenous IL-6 (0-5000 pg/ml) or IL-17A (0-5000 pg/ml), and rituximab was added 24 h before apoptosis detection. **d** Percentages of apoptotic SU-DHL-2 cells in 72-h cultures in the presence of rituximab (10 μg/ml), doxorubicin (2 μM) and Ara-C (2 μM). SU-DHL-2 cells were pre-cultured with exogenous IL-6 (0.5 ng/ml) and IL-17A (0.1 ng/ml) for 48 h before addition of drugs (rituximab, doxorubicin and Ara-C). **e** and **f** The sum of apoptotic SU-DHL-4 cell percentages induced spontaneously or by rituximab (10 μg/ml) in all groups. The culture conditions were the same as those in (**a**). **g** Representative FACS dot plots of apoptotic SU-DHL-4 cell percentages induced spontaneously or by rituximab (10 μg/ml) in all groups. The data shown represent one of three independent experiments. Error bars represent SD. Significance was determined using one-way ANOVA. (*P < 0.05; **P < 0.01)

Published data indicate that IL-6 or IL-17A acts as a pro-tumor factor in MCL or DLBCL[12, 16]. Based on these data and the present results, we hypothesized that hBMSCs secrete IL-6 into the TME of DLBCL, and IL-6 directly promotes DLBCL growth and induces Th17 cells to secrete IL-17A, which increases this promoting effect. To verify this hypothesis, hBMSCs or exogenous IL-6 and/or IL-17A were co-cultured with SU-DHL-4 cells to induce proliferation, and then exogenous aIL-6 or aIL-17A were used to block these effects. hBMSCs and exogenous IL-6 and IL-17A promoted SU-DHL-4 cell proliferation, and aIL-6 or aIL-17A abolished these effects (Fig. 5a). IL-17A increased the promoting effects of IL-6, whereas aIL-6 combined with aIL-17A abolished these effects (Fig. 5a).

Next, we examined the effect of exogenous IL-6 and/or IL-17A on spontaneous or drug-induced apoptosis in DLBCL cells. Exogenous IL-6 or IL-17A were added at increasing concentrations into SU-DHL-2 or SU-DHL-4 cell co-cultures for 48 h, followed by rituximab treatment for 24 h before the detection of apoptosis by FACS. The results showed that IL-6 or IL-17A markedly protected SU-DHL-2 or SU-DHL-4 cells from rituximab-induced apoptosis in a concentration-dependent manner (Fig. 5b and c). Based on these data, we determined the most appropriate concentrations of IL-6 and IL-17A for the next anti-apoptosis experiments as 500 pg/ml and 50 pg/ml, respectively. Then, the effects of IL-6 and IL-17A on protecting SU-DHL-2 cells against rituximab-, doxorubicin-, and Ara-C-induced apoptosis were examined. As shown in Fig. 5d, IL-6 significantly decreased the percentage of apoptotic cells in the three groups, whereas IL-17A further enhanced these effects.

Finally, to further confirm the effects of IL-6 and/or IL-17A on DLBCL cell apoptosis, IL-6 and/or IL-17A neutralizing antibodies were used to block IL-6 and IL-17A mediated signaling. The results showed that hBMSCs, IL-6, or IL-17A decreased spontaneous or rituximab-induced apoptosis in SU-DHL-4 cells, and aIL-6 or aIL-17A abolished these effects (Fig. 5e and f). In addition, IL-17A could reinforce the protective effects of IL-6, and aIL-6 with aIL-17A abolished these effects (Fig. 5e and f). Figure 5g shows representative FACS plots of spontaneous or rituximab-induced apoptosis of SU-DHL-4 cells.

### IL-6 or hBMSCs promote DLBCL cell growth via the JAK2/STAT3 signaling pathway

**Fig. 6:**
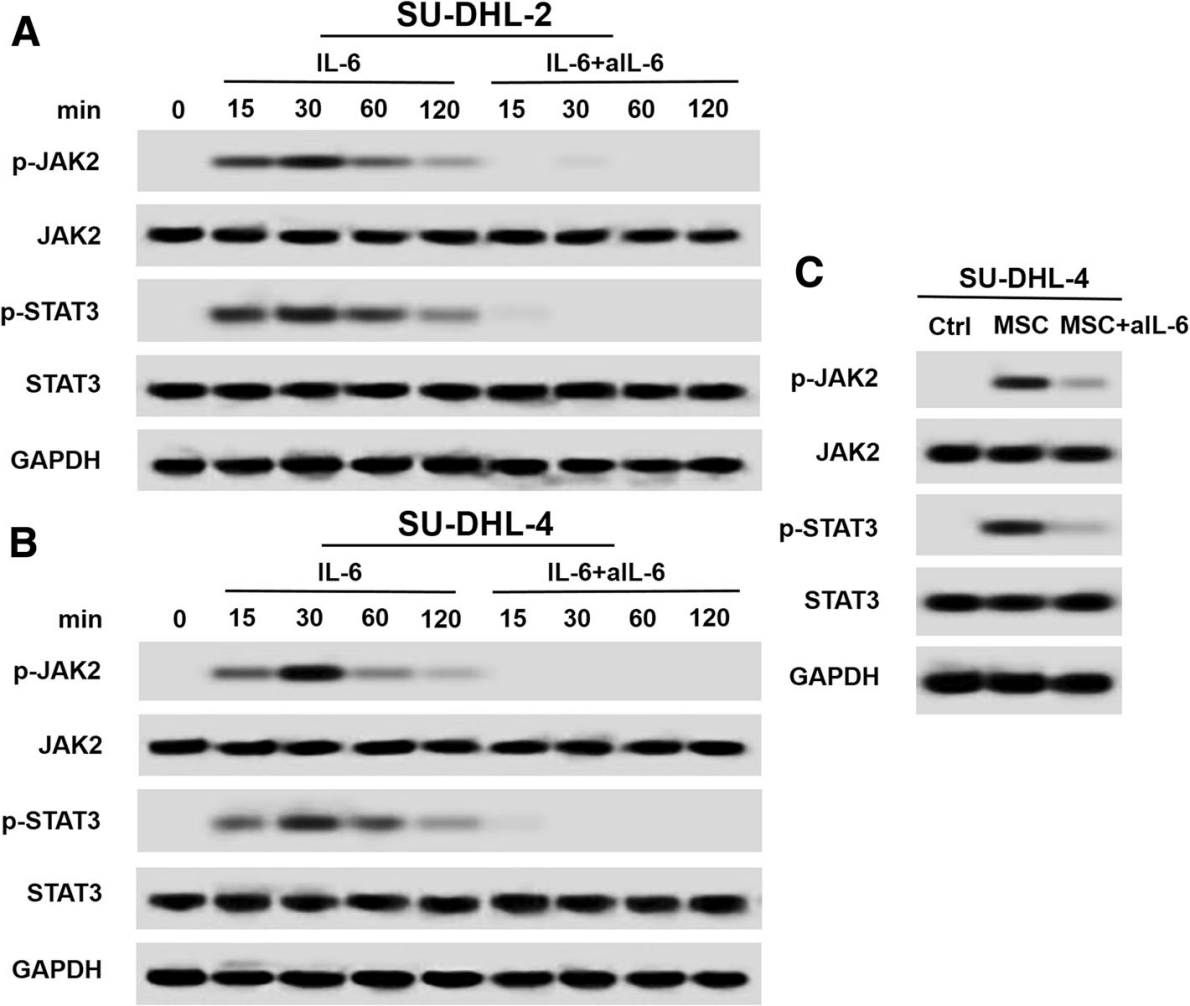
JAK2 and STAT3 phosphorylation were involved in IL-6- or hBMSCs-mediated DLBCL cell growth. Western blot images showing JAK2 (**a**) and STAT3 (**b**) phosphorylation in SU-DHL-2 and SU-DHL-4 cells detected at 0, 15, 30, 60, and 120 min in cultures with IL-6 (0.5 ng/ml) and/or aIL-6 (50 μg/ml). **c** Western blot images showing JAK2 and STAT3 phosphorylation in SU-DHL-4 cells pretreated without or with hBMSCs (1:1) and/or aIL-6 (50 μg/ml) for 30 min. The data shown represent one of three independent experiments

To investigate the signaling pathways involved in the effects of hBMSCs or IL-6, JAK2 and STAT3 phosphorylation was assessed in DLBCL cells. As shown in Fig. 6a and b, IL-6 significantly promoted the phosphorylation of JAK2 and STAT3 in both SU-DHL-2 and SU-DHL-4 cells, whereas IL-6-neutralizing antibodies abrogated IL-6-induced phosphorylation of JAK2 and STAT3. Similarly, hBMSCs upregulated p-JAK2 and p-STAT3 in SU-DHL-4 cells, whereas IL-6-neutralizing antibodies abolished these effects (Fig. 6c). Taken together, these results supported that IL-6 or hBMSCs promoted DLBCL cell growth via the JAK2/STAT3 signaling pathway.

### IL-17A promotes DLBCL cell growth by upregulating cyclin D2 via the PI3K/Akt signaling pathway

To further examine the molecular mechanisms underlying IL-17A-mediated DLBCL cell growth, microarray and bioinformatics analyses were performed together with qPCR and western blotting to assess the expression of related genes and proteins. Three samples of SU-DHL-2 cells were co-cultured with IL-17A for 72 h and an additional three samples were cultured without IL-17A as controls. The six samples of SU-DHL-2 cells were subjected to microarray analysis.

**Fig. 7:**
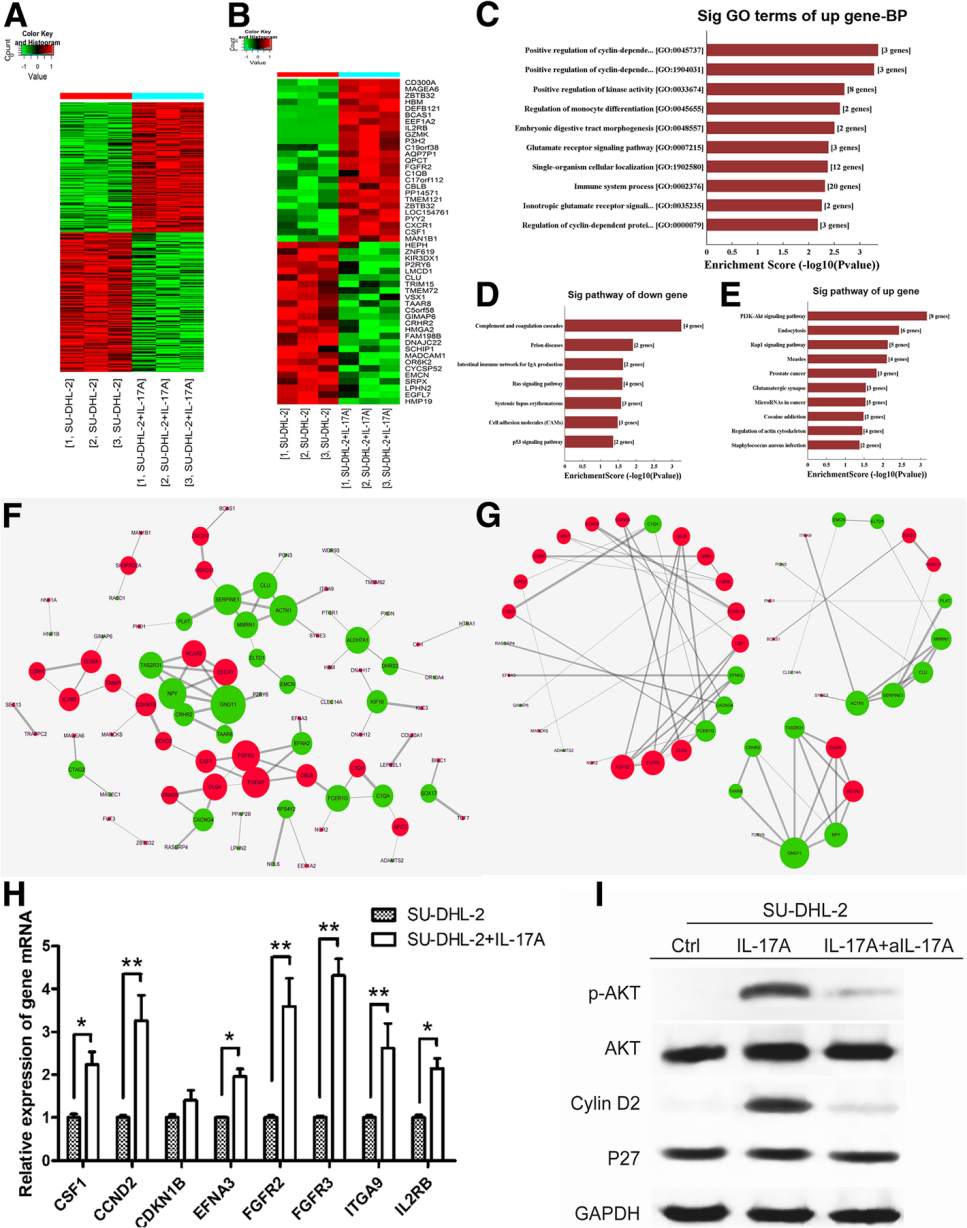
Analysis of gene expression profiles in IL-17A-treated SU-DHL-2 cells. **a** Heat map of all DEGs after IL-17A treatment. **b** Heat map of the top 25 most significant up- and down-regulated DEGs ranked by fold change. **c** The most significant GO biological process terms of upregulated DEGs. **d** and **e** The most significant pathways of down- or up-regulated DEGs based on the KEGG database. The co-expression networks of all DEGs (**f**) and part of DEGs (**g**) were analyzed using the STRING database and Cytoscape 3.6.0 software. Node size represents the degree of each gene, and the green or red nodes represent down- or up-regulated genes, respectively. Edge size represents the combined score. **h** Graph of the relative mRNA levels of eight upregulated DEGs in the PI3K/Akt in SU-DHL-2 cells without or with IL-17A (200 pg/ml) co-cultured for 72 h. **i** Western blot images showing Akt, p-Akt, cyclin D2, and P27 in SU-DHL-2 cells without or with IL-17A (200 pg/ml) co-cultured for 72 h. Error bars represent SD. Significance was determined using two-tailed independent-sample Student’s t/t′ test. (*P < 0.05; **P < 0.01, compared with the SU-DHL-2 group)

As shown in Figs. 7, 288 DEGs were identified (139 upregulated and 149 downregulated), and the heat map is shown in Fig. 7a. The heat map of the top 25 most significant up- and down-regulated DEGs ranked by fold change is shown in Fig. 7b. The top 10 most enriched biological process GO terms of all upregulated DEGs were positive regulation of cyclin-dependent protein serine/threonine kinase activity, positive regulation of cyclin-dependent protein kinase activity, regulation of cyclin-dependent protein serine/threonine kinase activity, and other terms (Fig. 7c). Three of these terms were related to cell cycle regulation, and the involved upregulated DEGs were PKD1, CDKN1B, and CCND2. Based on KEGG database pathway analysis, the most enriched pathways of downregulated DEGs included the Ras signaling pathway and p53 signaling pathway (Fig. 7d). The most enriched pathways of upregulated DEGs included the PI3K/Akt signaling pathway, Rap1 signaling pathway, prostate cancer, and microRNAs in cancer (Fig. 7e). These pathways were all connected to tumor growth, and the PI3K/Akt signaling pathway (eight involved genes: CCND2, CDKN1B, CSF1, EFNA3, FGFR2, FGFR3, IL2RB, and ITGA9) ranked first among upregulated pathways (Enrichment Score = 3.159187). The STRING database and Cytoscape 3.6.0 software were used to generate co-expression networks of all interacting DEGs to identify the genes that may play important roles in IL-17A-mediated SU-DHL-2 cell growth (Fig. 7f and g). Node size represented the degree of expression of each gene; green or red nodes represented down- or up-regulated genes, respectively, and edge size represented the combined score. We found that the eight genes involved in the PI3K/Akt signaling pathway graded high in the gene co-expression networks (Table 3), suggesting that the PI3K/Akt signaling pathway plays an important role in IL-17A-mediated SU-DHL-2 cell growth.

**Table 3:** 40 highest degree nodes in the DEGs co-expression networks

Nodes	Degree	Up or Down
GNG11	7	down
FGFR3	5	up
FGFR2	5	up
NPY	5	down
ACTN1	5	down
SERPINE1	5	down
ALDH7A1	4	down
TAS2R31	4	down
FCER1G	4	down
CXCR1	4	up
DLG4	4	up
HCAR2	4	up
CLU	4	down
MMRN1	4	down
IL2RB	3	up
CSF1	3	up
GZMA	3	up
CACNG4	3	down
CBLB	3	up
CRHR2	3	down
EFNA2	3	down
KIF19	3	down
CDKN1B	3	up
C1QA	3	down
APCS	2	up
SH3PXD2A	2	up
TXNIP	2	up
EMCN	2	down
ELTD1	2	down
DHRS3	2	down
CTAG2	2	down
GZMK	2	up
GRIN3B	2	up
SOX17	2	down
TAAR8	2	down
HDAC11	2	up
ZNF217	2	up
RPS4Y2	2	down
CCND2	2	up
C1QB	2	up

Finally, the mRNA expression of the eight genes was measured by qPCR, and PI3K/Akt pathway-related protein expression was assessed by western blotting. Consistent with the microarray results, the relative mRNA expressions of CSF1, CCND2, EFNA3, FGFR2, FGFR3, IL2RB, and ITGA9 were significantly higher in IL-17A-treated SU-DHL-2 cells (Fig. 7h). Cyclin D2 is encoded by the CCND2 gene, which is a downstream molecule of the PI3K/Akt pathway and regulates the cell cycle. IL-17A upregulated p-Akt and cyclin D2 expression in SU-DHL-2 cells, whereas aIL-17A abrogated these effects (Fig. 7i). However, IL-17A and aIL-17A had no effect on P27 expression in SU-DHL-2 cells. In summary, the results indicated that IL-17A promoted the growth of SU-DHL-2 cells by upregulating cyclin D2 via the activation of PI3K/Akt signaling.

## Discussion

In the present study, we demonstrated that hBMSCs promoted the growth of DLBCL not only by secreting IL-6 into the TME both in vitro and *invivo*, but also by inducing Th17 cell differentiation, thereby increasing IL-17A levels in vitro. IL-6 and IL-17A synergistically promoted the growth and drug-resistance of DLBCL cells by protecting them from spontaneous or drug-induced apoptosis in vitro. IL-6 promoted DLBCL cell growth via the JAK2/STAT3 pathway, whereas IL-17A promoted the growth of DLBCL cells by upregulating cyclin D2 through the activation of PI3K/Akt signaling.

Tumor cells are protected by their TME, and MSCs are important components of the TME in NHL. Whether MSCs are a pro-tumor or anti-tumor factor in the development of NHL remains controversial. A previous study showed that hBMSCs inhibit the growth of EBV-Burkitt-type BJAB cells and EBV+ B lymphoblastoid SKW6.4 cells in nude-SCID mice [23]. In another study, human adipose tissue-derived MSCs (hATMSCs) inhibited EL4 murine T-cell lymphoma growth in vitro and in vivo [24]. However, accumulating evidence indicates that MSCs act as pro-tumor factors that interact with lymphoma cells and promote lymphoma growth, survival, and drug-resistance through multiple mechanisms [8, 25]. Recent studies show that hATMSCs or hBMSCs promote growth and drug-resistance in Burkitt lymphoma cells or DLBCL cells in vitro or in vivo [9, 10, 26, 27]. Here, we found that hBMSCs promoted the growth and drug-resistance of SU-DHL-2 and SU-DHL-4 cells in vitro and in vivo*.* As MSCs are a heterogeneous population of activated fibroblasts derived from various tissues, different tissue-derived MSCs may have distinct effects on the growth of different types or stages of NHL.

Research on the role of the TME in DLBCL pathogenesis suggests that there are three types of DLBCL drug-resistance: de novo (TME-mediated) drug-resistance, acquired drug-resistance (chronic exposure), and DLBCL adherent to stromal cells [28]. We previously demonstrated that IL-17A in the TME induces irradiation or rituximab resistance in DLBCL.[17–19]. In the present study, we further elucidated de novo TME-mediated resistance and identified the signaling pathways (JAK2/STAT3 and PI3K/Akt) involved in DLBCL. HBMSCs secreted cytokines into the TME and created pro-survival conditions for DLBCL cells, eventually inducing drug-resistance.

The cytokines and immune cells in the TME play a vital role in the development of DLBCL [29]. Numerous researchers have demonstrated that MSCs facilitate lymphoma growth by secreting pro-tumor cytokines (such as IL-6 and IL-10), inducing angiogenesis, promoting epithelial and mesenchymal transition, and inhibiting apoptosis of tumor cells [25]. However, little is known about the role and mechanisms by which hBMSCs modulateTh17 and Treg cell differentiation and the levels of related cytokines in the TME of DLBCL. Our results showed that hBMSCs simultaneously secreted IL-6 and induced Th17 cells to secrete IL-17A in the TME of DLBCL. This suggests a dual effect of hBMSCs on promoting DLBCL progression and drug-resistance.

Several types of cytokines in the TME can facilitate the growth of tumor cells. IL-6 is a key cytokine in the TME that is secreted by many cells, such as malignant cells and MSCs. Many recent studies showed that IL-6 plays a pivotal role in cancer development, chemoresistance, and cancer stem cell maintenance [30]. IL-6 promotes the growth and drug-resistance of MCL [12], and high levels of IL-6 in the peripheral blood of DLBCL patients indicates a poor prognosis [13, 14]. IL-17A is another important cytokine in the TME that is mainly secreted by Th17 cells. IL-6 acts together with TGF-β to promote Th17 cell differentiation and IL-17A secretion by upregulating RORγt expression. IL-17A promotes the growth of human germinal center-derived DLBCL in vitro and in mice by inducing angiogenesis [16]. In previous work, we showed that IL-6 upregulated IL-17A by inducing Th17 or Foxp3+ Treg cell differentiation in vitro or in the peripheral blood of DLBCL patients, thereby promoting the growth of k1106 cells or SU-DHL-4 cells by inhibiting irradiation- or rituximab-induce apoptosis through the suppression of p53 expression [17–19]. Consistent with previous data, the present results indicated that hBMSCs simultaneously secreted IL-6 and induced Th17 cells to secrete IL-17A into the TME; IL-6 and IL-17A synergistically promoted the growth of SU-DHL-2 and SU-DHL-4 cells and drug-resistance. In addition, we showed that hBMSCs induced PBMC differentiation into Treg cells and increased the level of TGF-β in the TME in vitro. Based on our previous studies, we speculated that hBMSCs induced Treg cells to secrete IL-17, generating IL-17 + Treg cells [17–19]. Published data indicate that Treg cells play an important role in the pathogenesis of cancer. Wang et al. [31] reported that Treg cells suppress tumor-specific CD8+ T cells, thus weakening the anti-cancer capacity of the body. Previous data from our group show that Treg cells are another source of IL-17 in the TME that are involved in DLBCL survival and growth [17–19]. This evidence supports the new “dual effects” mechanism underlying the role of hBMSCs in promoting DLBCL progression and drug-resistance.

To further investigate the signaling pathways involved in the function of IL-6 and IL-17A in DLBCL cells, we performed microarray analysis, western blotting, and qPCR. Recent studies from our group showed that IL-17A promotes DLBCL cell growth by suppressing p53 expression [17, 18]. In the present study, microarray analysis showed that IL-17A decreased the activity of the p53 pathway (Fig. 6d). In addition, IL-6 or IL-17A promoted DLBCL cell growth via the JAK2/STAT3 pathway or by upregulating cyclin D2 via the PI3K/Akt pathway.IL-6 generally binds to membrane IL-6 receptor and gp130, activating the JAK2/STAT3 pathway in target cells [32]. JAK2/STAT3 is a key pathway in the pathogenesis of NHL, and activation of this pathway is closely related to increased chemoresistance and apoptosis inhibition in NHL [33, 34]. Therefore, STAT3 inhibitors have great potential as novel targeted agents in NHL [35], and early-stage studies of STAT3 inhibitors have shown encouraging results in NHL treatment [36, 37].

The PI3K/Akt signaling pathway is a crucial pathway involved in the regulation of the proliferation and survival of tumor cells in various types of malignancies, including DLBCL [38].The PI3K/Akt pathway is highly activated in DLBCL and induces a survival advantage, increasing the metastatic ability and drug-resistance [39]. Here, we showed that IL-17A upregulated p-Akt and cyclin D2 expression in SU-DHL-2 cells. Akt is a serine-threonine kinase that is also known as protein kinase B, one of the major oncogenic effectors of the PI3K/Akt pathway [40]. Akt is activated by phosphatidylinositol (3,4,5)-triphosphate and is phosphorylated to become p-Akt, which negatively regulates the functions of several downstream pathways [40], and cyclin D2 is one of these downstream molecules. Cyclin D2 is one of the D-type cyclins (D1, D2, and D3), which positively regulate the cell cycle and mediate the pathogenesis of some lymphomas [41]. Overexpression of cyclin D1 is a hallmark of MCL, whereas cyclins D2 or D3 are not as specific to certain lymphomas as cyclin D1 [42]. Cyclin D2 is overexpressed in cyclin-D1-negative MCL, chronic lymphocytic leukemia/small lymphocytic lymphoma, and DLBCL [42–44]. Furthermore, cyclin D2 overexpression is negatively correlated with the prognosis of DLBCL [45, 46]. Therefore, our data indicate that p-Akt or cyclin D2 may be a potential molecular target for the treatment of refractory or chemoresistant DLBCL.

Many studies showed that MSCs migrate into the TME of various tumors [24, 47, 48]. A recent study showed that radiation induces tumor cells to release SDF-1α and platelet-derived growth factor-β, which bind to CXCR4 and platelet-derived growth factor receptor-βon circulating BMSCs, respectively; this results in the migration of BMSCs into the TME and promotes tumor growth [49]. CXCR4, an α-chemokine receptor specific for SDF-1α, is expressed in various tumors and linked to metastasis to tissues containing a high concentration of SDF-1α [50]. CXCR4 expression is also detected in malignant lymphoma cell lines, and its inhibition by a monoclonal antibody enhances apoptosis, decreases proliferation, and inhibits migration [51]. Reinholdt et al. reported that the CXCR4 antagonist plerixafor synergistically enhances the anti-proliferative/pro-apoptotic effect of rituximab on DLBCL cells [52]. Lapa et al. demonstrated that CXCR4-directed radioligand therapy in DLBCL is feasible [53]. Furthermore, a recent study shows that CXCR4 expression may serve as a biomarker for the prognosis of DLBCL and for the development of new therapeutic strategies [54]. Therefore, MSC migration and maturation could be abrogated using CXCR4 antagonists and tyrosine kinase inhibitors [55].

## Conclusions

In conclusion, in the present study, hBMSCs promoted the growth of DLBCL by secreting IL-6 both in vitro and in vivo and elevating IL-17A levels in the TME in vitro. IL-6 and IL-17A synergistically promoted the growth and drug-resistance of DLBCL cells by protecting them from spontaneous or drug-induced apoptosis in vitro. IL-6 or IL-17A activated the JAK2/STAT3 pathway or upregulated cyclin D2 through the activation of PI3K/Akt signaling in vitro*,* respectively. The present results indicated that hBMSCs may have a “dual effect” on promoting DLBCL progression and drug-resistance by secreting IL-6 and elevating IL-17A levels. IL-6, IL-17A, p-STAT3, p-Akt, or cyclin D2 may be potential molecular targets for overcoming drug-resistance in patients with relapsed or refractory DLBCL.

## Abbreviations

DLBCL: Diffuse large B cell lymphoma
HBMSCs: Human bone marrow-derived mesenchymal stem cells
IL: Interleukin
NHL: Non-Hodgkin’s lymphoma
PBMCs: Peripheral blood mononuclear cells
Th17: T helper 17
TME: Tumor microenvironment
Treg: Regulatory T
